# 540. Spray-on Local Anesthetic for Burns? Nearly! A First in Man Study in Suturing Wounds

**DOI:** 10.1093/jbcr/irag033.237

**Published:** 2026-04-06

**Authors:** Steven Jeffery

**Affiliations:** 202 Multi Role Medical Regiment, Bentley Pauncefoot, Nr Bromsgr, England

## Abstract

**Introduction:**

Pain management is one of the most important aspects of treating partial thickness burns. Current techniques involve systemic analgesia, which has consequences for the patient and for the hospital. Optimal analgesia often results in an obtunded patient. A widely used veterinarian product contains Lidocaine, Bupivacaine, Adrenaline and Cetrimide. We are working on bringing this spray-on product to the human world. This study is the first in man trial, looking at safety and efficacy.

**Methods:**

24 adults with small-to-medium lacerations of the limbs were treated with in four cohorts with Local Anesthesia/Epinephrine Spray (LAES) prior to suturing. Cohort 1 had LAES applied without a cover. Cohort 2 had an occlusive cover applied and left for 5 minutes. Cohort 3 were left for 10 minutes and Cohort 4 for 15 minutes. Blood levels were taken for toxicology and pain scores were assessed. For suturing, 'rescue' analgesia with injectable LA was available if required.

**Results:**

None of the cases treated with an occlusive cover (Cohorts 2-4) required rescue analgesia and had pain scores between 0 and 2. One patient in Cohort 1 required rescue analgesia. There were no significant changes in blood pressure, ECG, heart rate or respiratory rate. There were no neurological symptoms. Blood lidocaine levels peaked at 5 nanograms/ml (toxicity not normally described until 6 micrograms/ml). Bupivacaine levels peaked at 10.8 nanograms/ml (toxicity not normally described until 2 micrograms/ml).

**Conclusions:**

The application of LAES with an occlusive cover offers sufficient analgesia after 5 minutes to allow suturing of small-to-medium lacerations in adults.

**Applicability of Research to Practice:**

We are now looking at extending our trials and would like to undertake a burn trial in particular. Please come and speak to me during the conference if you would like to be involved.

**Funding for the study:**

N/A.

